# Altered Hemodynamics in the Embryonic Heart Affects Outflow Valve Development

**DOI:** 10.3390/jcdd2020108

**Published:** 2015-05-15

**Authors:** Vinal Menon, John F. Eberth, Richard L. Goodwin, Jay D. Potts

**Affiliations:** 1 Department of Cell Biology and Anatomy, School of Medicine, University of South Carolina, Columbia, SC 29209, USA; E-Mails: vinal.menon@uscmed.sc.edu (V.M.); john.eberth@uscmed.sc.edu (J.F.E.); 2 Biomedical Engineering Program, College of Engineering and Computing, University of South Carolina, Columbia, SC 29208, USA; 3 Biomedical Sciences, School of Medicine, University of South Carolina, Greenville, SC 29605, USA; E-Mail: goodwirl@greenvillemed.sc.edu

**Keywords:** valvular disease, cardiac valve development, endocardial cushions, EMT, hemodynamics

## Abstract

Cardiac valve structure and function are primarily determined during early development. Consequently, abnormally-formed heart valves are the most common type of congenital heart defects. Several adult valve diseases can be backtracked to abnormal valve development, making it imperative to completely understand the process and regulation of heart valve development. Epithelial-to-mesenchymal transition (EMT) plays an important role in the development of heart valves. Though hemodynamics is vital to valve development, its role in regulating EMT is still unknown. In this study, intracardiac hemodynamics were altered by constricting the outflow tract (OFT)/ventricle junction (OVJ) of HH16–17 (Hamilton and Hamburger (HH) Stage 16–17) chicken embryos, *ex ovo* for 24 h. The constriction created an increase in peak and time-averaged centerline velocity along the OFT without changes to volumetric flow or heart rate. Computational fluid dynamics was used to estimate the level of increased spatially-averaged wall shear stresses on the OFT cushion from AMIRA reconstructions. OFT constriction led to a significant decrease in OFT cushion volume and the number of invaded mesenchyme in the OFT cushion. qPCR analysis revealed altered mRNA expression of a representative panel of genes, vital to valve development, in the OFT cushions from banded hearts. This study indicates the importance of hemodynamics in valve development.

## 1. Introduction

Valves in healthy adult four-chambered hearts allow for unidirectional flow of blood to both the pulmonary and systemic circuits. The structure and function of these cardiac valves are primarily determined during early embryonic development. Consequently, abnormally-formed heart valves, especially defective outflow valves, are the most common type of congenital heart defects (CHDs) [1]. Abnormal cardiac valve structure may be present at birth; however, this may be asymptomatic and could predispose the individual to valve disease later in life. Thus, several adult valve diseases may be backtracked to abnormal valve development. This makes it imperative to understand the molecular basis of heart valve development and the effect that hemodynamics has on these processes.

The heart is the first organ to develop and function during embryogenesis. It initially forms as a tubular blood vessel comprised of endocardial endothelial cells surrounded by myocardium [2]. An acellular layer of extracellular matrix (ECM), known as cardiac jelly, separates the two heart tube layers [3]. Myocardial cells secrete most of the ECM, which is composed primarily of glycosaminoglycans, chondroitin sulfate and hyaluronan [4]. After the onset of rightward looping, the cardiac jelly expands at two distinct foci—the atrio-ventricular (AV) canal and the outflow tract (OFT)—forming endocardial cushions (valve primordia) that eventually develop into the AV and OFT valves [3,5]. The mechanisms of cushion development are conserved in both the AV canal and the OFT. In addition to the endocardially-derived cells, neural crest cells also contribute to the OFT valves [2].

The regulated process of epithelial-to-mesenchymal transition (EMT) plays a crucial role in embryonic differentiation and development [6]. In response to local stimuli, a subset of AV and OFT endocardial cells delaminate into the cardiac jelly, due to loss of cell-cell contact, and adopt a migratory, mesenchymal phenotype leading to the onset of EMT [5]. In an *in vitro* model, it was shown that chick AV canal explants (consisting of endocardial cushion cells and myocardium) cultured on collagen gels exhibit EMT [7]. Furthermore, it has been shown that only the endothelium of the AV canal and OFT, but not that of the ventricle, undergoes EMT to produce the mesenchyme, thus demonstrating a requirement for specialized spatial signaling to induce EMT [8,9].

As blood flows through a vessel, shear stress is produced due to friction between the flowing blood and the vessel wall [10]. It is known that endothelial cells sense and respond to hemodynamics by means of mechanotransducer proteins and initiate the rearrangement of the cytoskeleton, leading to these cells being aligned parallel to the direction of flow [10]. Hemodynamics has been shown to play an important role in regulating embryonic cardiogenesis [11,12]. We have previously shown that in an *in vitro* 3D culture system, the expression and deposition of fibrous ECM proteins in AV and OFT cushions were affected by shear stress [13,14]. However, it is not clearly understood how hemodynamics regulates (initiates and eventually terminates) EMT during heart valve development. The purpose of this study was to alter the hemodynamics in the embryonic chicken heart by constricting the OFT and to study the effects of this intervention on early developmental processes. We hypothesize that changes in hemodynamics influence both EMT and OFT valve development, resulting in altered ECM production.

## 2. Materials and Methods

### 2.1. OFT Banding to alter Intracardiac Hemodynamics

Fertilized Bovan chicken eggs (Clemson University, Clemson, SC, USA) were incubated in a rocker incubator at 37 °C till they reached Hamilton and Hamburger (HH) Stage 17. After sterilizing the outside of the egg with 70% ethanol, the shell was gently cracked with a scalpel handle, and the embryo, positioned on top of the yolk, was then gently transferred to a petri dish containing Tyrode’s buffer (Sigma, St. Louis, MO, USA) supplemented with sodium bicarbonate (1 g/L). The OFT was banded (constricted), *ex ovo*, using a 10-0 nylon suture at the OFT/ventricle junction (OVJ). Control embryos were also transferred to a Tyrode’s-containing petri dish, but were not subjected to the banding intervention. Banded and control embryos were then placed in a humidified incubator at 37 °C for 24 h.

### 2.2. Analysis of Change in Flow Velocity

Changes in blood flow velocity at the OVJ of banded (*n* = 5) and control (*n* = 5) embryos were determined by ultrasound using the Vevo 770 imaging system (VisualSonics, Toronto, ON, Canada). The petri dish containing the embryo to be imaged was placed on a heating pad and carefully filled to the brim with Tyrode’s buffer, making sure that the yolk sac was intact. A 708 scan head, operating in B mode, was used to obtain a 2D image of the beating heart, and the stage was moved till the OVJ was clearly visible. The pulsed-wave (PW) mode, at a pulse repetition frequency of 20 kHz, was used to obtain a waveform. For each embryo, peak velocities at the OVJ were measured for ten heartbeats. From the resulting data, the heart rate in beats-per-minute (BPM) was calculated by measuring the foot-to-foot time of the velocity wave, so that heart rate (HR) = 60/T. Time-dependent centerline and spatially-averaged velocities were measured from the PW Doppler waveforms using built-in software algorithms, and these waveforms were digitized using the ImageJ figure calibration plugin, so that the time-averaged and peak velocities could be found [15]. Embryos with a slowing heart rate during the test were not used for analysis. Embryos subjected to ultrasound imaging were not used for any other experiment.

### 2.3. Three-Dimensional OFT Reconstruction and Determination of the Extent of EMT

Banded (*n* = 3) and control (*n* = 3) embryos were carefully excised from the yolk sac and fixed overnight in 2% paraformaldehyde. Extra embryonic membranes were left intact, so as to prevent sectioning artifacts. Fixed embryos were dehydrated through an alcohol series, cleared with xylene, and penetrated with and embedded in paraffin. Five-micrometer serial sections were obtained on a microtome and stained with hematoxylin and eosin (H&E). Individual nuclei were counted from the H&E images of banded and control OFTs to determine the number of cells that invaded the cushions by EMT. TIFF images of the complete OFT were captured using a Nikon Optiphot-2 light microscope at a total magnification of 40× and loaded into the AMIRA software package (FEI Visualization Science Group, Burlington, MA, USA). All sections were aligned, and the OFT and myocardium was segmented. A 3D model of the OFT was then generated from which the volume of the OFT cushion was determined. Models were reduced to 2500 faces to improve manageability and exported as stereolithography (.stl) files to Geomagic Studio (3D Systems, Rock Hill, SC, USA) for smoothing and anatomical measurements of cross-sectional area and perimeter at the OVJ. These reconstructions were then converted into the Initial Graphics Exchange Specification (IGES) file format for finite element analysis.

### 2.4. Computational Fluid Dynamics

IGES files were imported as a continuous geometry into COMSOL Multiphisics 4.3b (Comsol, Burlington, MA, USA). In COMSOL, faces perpendicular to the axis of blood flow were generated at the proximal (ventricle) and distal (OFT) sides to act as inlet and outlet conditions. A single representative 3D model was generated for the control, and a single representative 3D model was generated for the banded hearts. From these models, the cross-sectional areas at the OVJ were measured using Geomagic (Control: A = 14,491; Banded: A = 1714 μm^2^). Unique volumetric flow inlet boundary conditions were applied for each of the control (*n* = 5) and banded hearts (*n* = 5). At a given cross-section, the time-averaged, peak and late retrograde flow velocities were calculated from the spatially-averaged velocity and used to calculate the inlet time-averaged, peak and late retrograde volumetric flow rates. A zero-pressure boundary condition was applied at the distal location for all samples. Despite the importance of a changing geometry during the cardiac cycle, our analyses are limited by the available imaging modalities to tissue arrested under physiological loading. Accordingly, a rigid wall assumption and no-slip boundary conditions were assigned for the remaining surfaces. Using a hematocrit of 19.4% for HH Stage 16–17 chick embryos, we assumed that blood, at high shear rates, behaved as a Newtonian fluid; therefore, the density ρ = 1025 kg/m^3^ and apparent dynamic viscosity μ = 0.0015 Pa·s remained constant in our simulations. Accordingly, the lowest time-averaged shear rate from our simulations was γ = 424 ± 113 s^−1^. Similarly, low peak Reynolds numbers (Re = 7.08 ± 1.06) indicated laminar flow behavior, while a rough estimate of the Womersley numbers (α = 0.18 ± 0.02), a metric used to relate pulsatile-inertial to viscous effects, yields a low value, as well. Collectively, these results suggest that quasi-static conditions are sufficient for analysis [16]. Wall shear stresses were calculated on the surface of the blood-tissue interfaces and spatially averaged for each of the flow conditions (time-averaged, peak and late cardiac cycle retrograde flow). The time-averaged pressure drop across the stenosis was calculated as the difference between the inlet and outlet (P = 0 mmHg) pressures.

### 2.5. Extraction of Total RNA from OFTs

OFTs (cushion + myocardium) were dissected after whole hearts were carefully excised from banded and control embryos. Each sample (*n* = 3–4) consisted of OFT tissue from about 20–22 pooled hearts. Total RNA was extracted using the GeneJET RNA purification kit (Thermo Scientific, Waltham, MA, USA) according to the manufacture’s protocol. RNA was eluted from the columns with 50 μL of nuclease-free water (provided in the kit). The concentration and purity of the eluted RNA samples were determined using the NanoPhotometer Pearl (Implemen, GmbH, Munich, Germany). RNA samples were stored at −80 °C until further analysis.

### 2.6. Complementary DNA Synthesis

cDNA was synthesized from total RNA using the iScript cDNA Synthesis Kit (BioRad, Hercules, CA, USA) according to the protocol provided. Five hundred nanograms of RNA were used in each reaction. Each cDNA sample was diluted 5× with nuclease-free water before being used for qPCR.

### 2.7. Quantitative Real-Time Polymerase Chain Reaction

**Table 1:** Primers used for qPCR.

Gene	Forward (F) and Reverse (R) Primers
*Arbp2*	F: 5′-GCTTTGCTTCGGTCTTTGAG-3′
	R: 5′-AACAACTTTCCGATCACCAC-3′
*klf2*	F: 5′-GCTTCTACCAGACAAACCCG-3′
	R: 5′-CAGGACTGGCCCATAACTGT-3′
*rhoA*	F: 5′-CAGCACCCTGCACTTGAGTA-3′
	R: 5′GCATCCTGTGAGTGCAGAAA-3′
*collagen 1α1*	F: 5′-TACCACTGCAAGAACAGCGT-3′
	R: 5′-TCGGTGACCCCATAGGTGAA-3′
*vinculin*	F: 5′-CAGGTAGTATCGGCTGCTCG-3′
	R: 5′-CCACCAGCCCTGTCATCTTT-3′
*elastin*	F: 5′-GTATCCCATCAAAGCTCCCA-3′
	R: 5′-CAGCTCCGTATTTAGCTGCC-3′
*periostin*	F: 5′-GGATGGTATGAGAGGATGTC-3′
	R: 5′GCAAAGAAAGTGAATGAACC-3′
*tenascinC*	F: 5′-AGGACACAGCCTCTGCAAGT-3′
	R: 5′-TACTGCCCCTGAGAGCTGAT-3′
*CDH11*	F: 5′-AAGACACTGGACCGAGAGGA-3′
	R: 5′-TTCTGAGGGCGGTTCCAAAG-3′
*filamin A*	F: 5′-CGGCGACTACACCATCAACA-3′
	R: 5′-GTCACTTTGGTGGGGTCGAA-3′
*TGFβRIII*	F: 5′-CTCTTACCGTCGTGGGCATT-3′
	R: 5′-CTGCTTCCCCTGTGTGAGAG-3′
*TGFβ2*	F: 5′-GAGAAAGCCAACCACAGAGC-3′
	R: 5′-GGTACAGCTCTATCCGCTGC-3′
*TGFβ3*	F: 5′-CACAATGAGTTGGGCATTTG-3′
	R: 5′-GGAACTCTGCTCGAAACAGG-3′
*snai2*	F: 5′-CACGCTCCTTCCTGGTCAAG-3′
	R: 5′-GGCTGCGGTATGATAGGGAC-3′
*has2*	F: 5′-CACCGCTGCTTACATTGTGG-3′
	R: 5′-TGTGATGCCAGGATAGCACC-3′
*mmp2*	F: 5′-TGATGATGACCGCAAGTGGG-3′
	R: 5′-TGTAGATCGGGGCCATGAGA-3′

qPCR was carried out using Fast SYBR Green Master Mix (Applied Biosystems, Foster City, CA, USA) on a BioRad CFX connect system. The following run conditions were used: enzyme activation at 95 °C for 3 min, denaturation at 95 °C for 10 s and annealing/extension at 60 °C for 30 s (40 cycles). A melt curve analysis was performed on each run. Each sample was run in triplicate, and qPCR was carried out on each gene three times. Relative gene expression was quantified using the Pfaffl method [17] with Arbp2 as the housekeeping (normalizing) gene. Primer sequences used for qPCR are listed in Table 1.

### 2.8 Statistical Analysis

Student’s *t*-test was used for analysis between groups, with α set to 0.05. All statistics and graphs were generated using Microsoft Excel 2011. Data in figures and in the text are reported as the means ± SD.

## 3. Results

### 3.1. Effect of OFT Banding on Blood Flow Velocity

**Figure 1:**
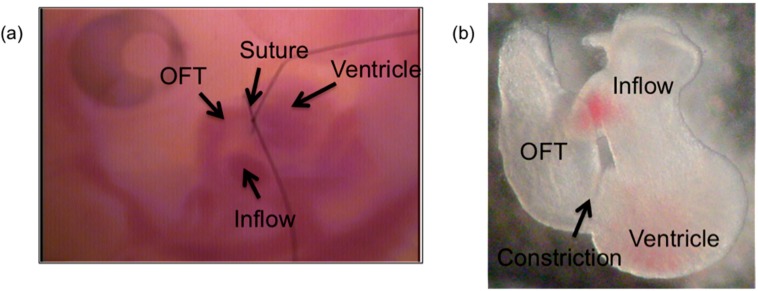
Our *in vivo* (ex ovo) chicken embryonic system. (**a**) Whole chicken embryo with the outflow tract (OFT) banded, imaged after 24 h; (**b**) isolated chick embryonic heart, 24 h after OFT banding.

Ultrasound imaging was performed to investigate whether the constriction of the OFT (Figure 1a,b) resulted in a change in flow velocity across the OVJ.

**Figure 2:**
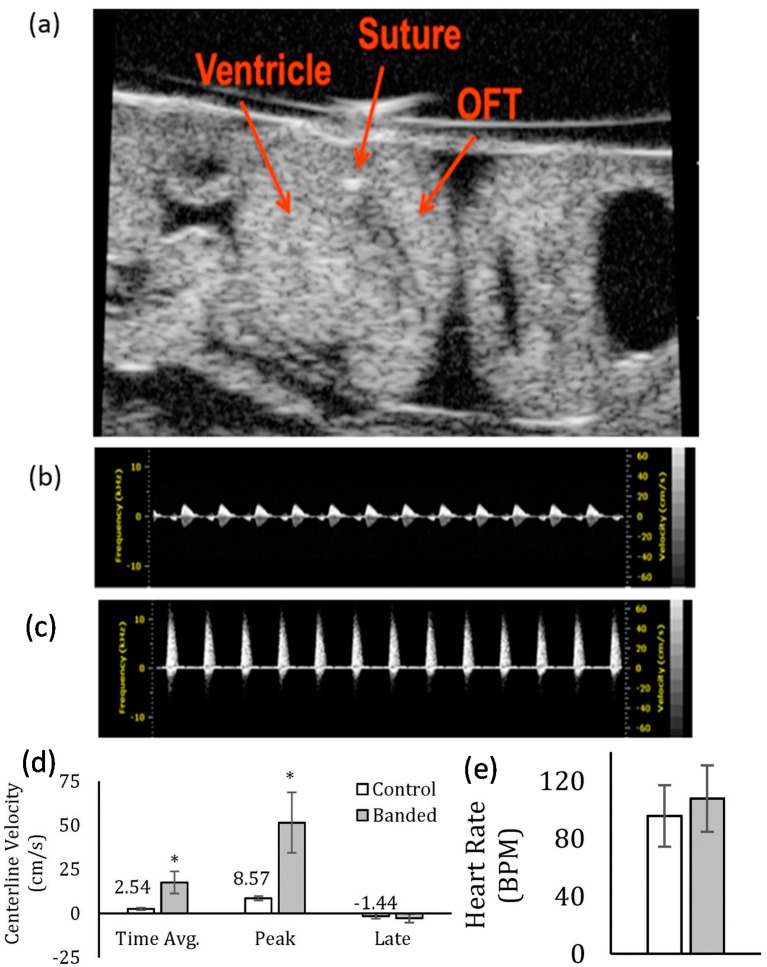
Ultrasound imaging. (**a**) B mode of a banded embryo showing the suture at the ventricle junction (OVJ). Pulsed-wave Doppler (PWD) at the OVJ for the (**b**) control and (**c**) banded hearts; (**d**) OFT banding caused significant changes in the time-averaged (avg) and peak centerline velocities, but not for velocities measured late in the cardiac cycle during retrograde flow measured at the OVJ; (**e**) the heart rate remained unchanged between controls and banded chick hearts. * *p* < 0.05.

The suture placed around the OVJ was clearly visible in a B-mode 2D image of the beating heart (Figure 2a). As expected, centerline flow velocity, measured with PW Doppler (Figure 2b,c), yielded higher time-averaged (control = 2.54 ± 0.68 cm/s, banded = 17.6 ± 6.32 cm/s; *p* = 0.002) and peak velocities (control = 8.57 ± 2.54 cm/s, banded = 51.5 ± 17.16 cm/s; *p* = 0.0018) at the banded OVJ relative to the control OVJ (Figure 2d). No statistical differences were found between heart rates of control (95.8 ± 21.4 BPM) and banded (108 ± 23.0 BPM) embryonic chick hearts (Figure 2e) (*p* = 0.19) or between the late cycle retrograde flow velocities (control = −1.44 ± 1.55 cm/s, banded = −2.74 ± 2.43 cm/s; *p* = 0.44) (Figure 2d).

### 3.2. Effect of OFT Banding on OFT Cushion Volume and Extent of EMT

**Figure 3:**
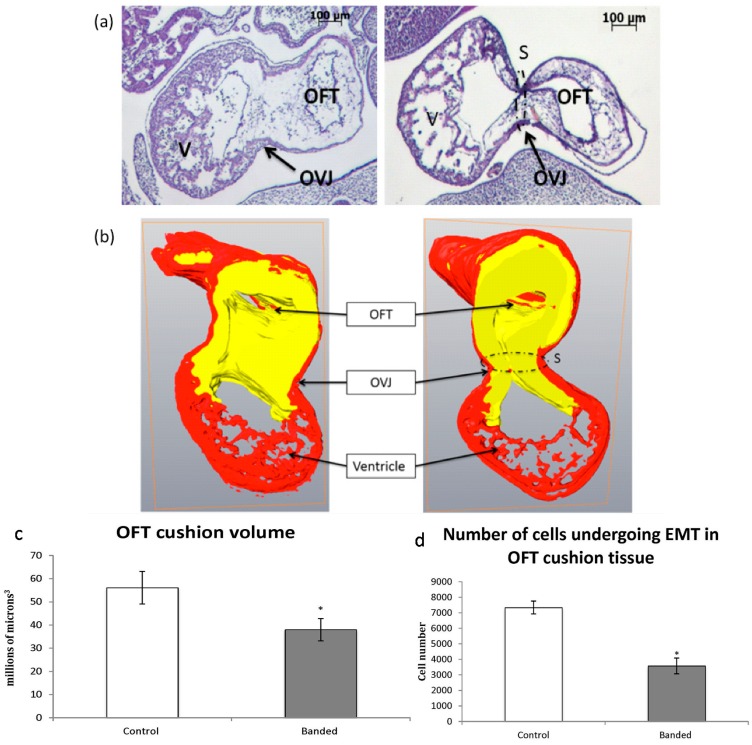
3D reconstructed OFT. (**a**) Representative H&E-stained images of a control (left) and banded OFT (right); (**b**) cut-away view of a control (left) and banded (right) 3D reconstructed OFT showing cushion (yellow) and myocardium (red). The effect of OFT banding on (**c**) OFT cushion volume and (**d**) the number of cells undergoing EMT. V, ventricle; S, position of suture. * *p* < 0.05.

To determine whether the alteration in hemodynamics in the embryonic heart caused any change in the volume of the OFT cushions, the OFTs of banded and control hearts were 3D reconstructed from H&E TIFF images of the OFTs (Figure 3a) using AMIRA (Figure 3b). A comparison of the AMIRA-generated volume data revealed a significant decrease in the OFT cushion volume of banded hearts compared to the control hearts (Figure 3c) (*p* = 0.01).

Individual cells that invaded the OFT cushions were counted from H&E images (Figure 3a), which revealed that OFT banding led to a significant decrease in the number of cells that entered the OFT cushion tissue, by means of EMT, in the banded relative to control OFT (Figure 3d) (*p* = 0.002).

### 3.3. Effect of OFT Banding on Computed Hemodynamics

**Figure 4:**
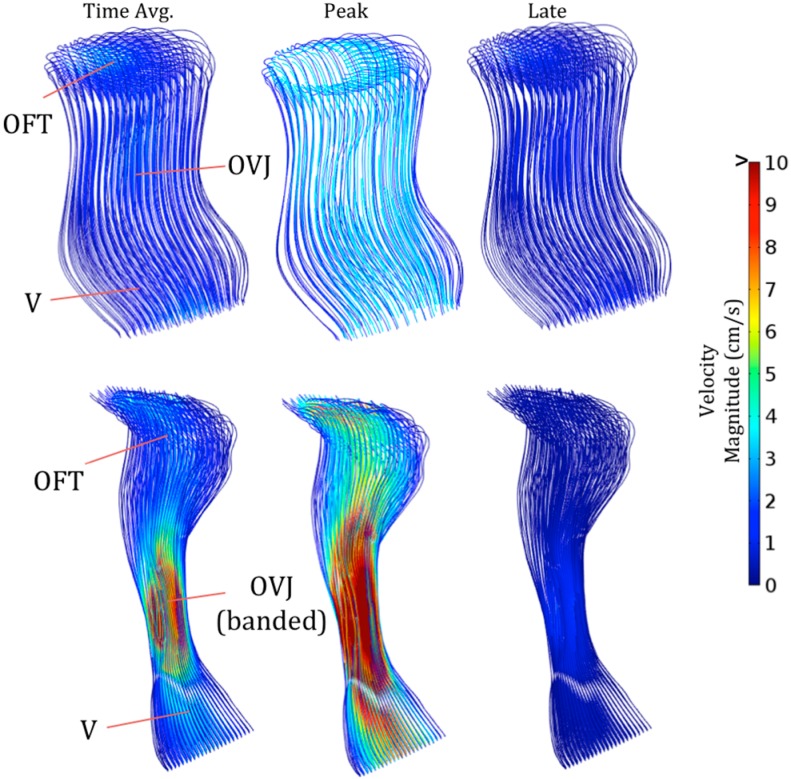
Velocity magnitude streamlines for (top) control chick hearts and (bottom) OFT banded chick hearts at the time-averaged flow velocity, peak flow conditions and late in the cardiac cycle during retrograde flow. V, ventricle; OVJ, OFT/ventricle junction; OFT, outflow tract.

**Figure 5:**
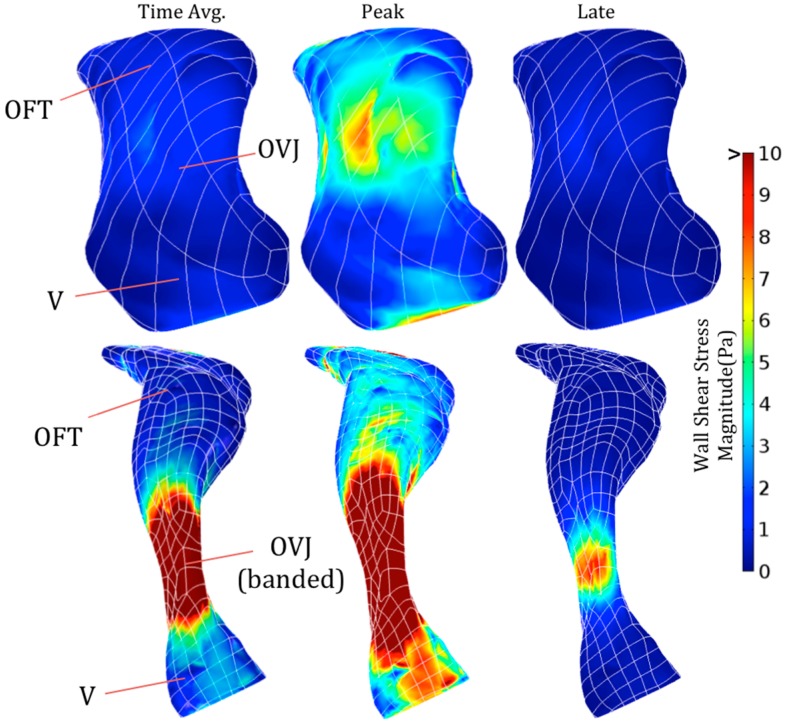
Wall shear stress magnitude for (top) control chick hearts and (bottom) OFT banded chick hearts at the time-averaged flow condition, during peak flow conditions and late in the cardiac cycle during retrograde flow. V, ventricle; OVJ, OFT/ventricle junction; OFT, outflow tract.

A 3D streamline velocity plot was generated from computational fluid dynamics (CFD) simulations to show the path and velocity magnitude within the developing embryonic chick heart. Consistent with the Doppler studies, the highest velocity occurred at the OVJ banding site (Figure 4). Consequently, this location experienced the highest time-averaged and peak wall shear stresses (Figure 5).

**Figure 6:**
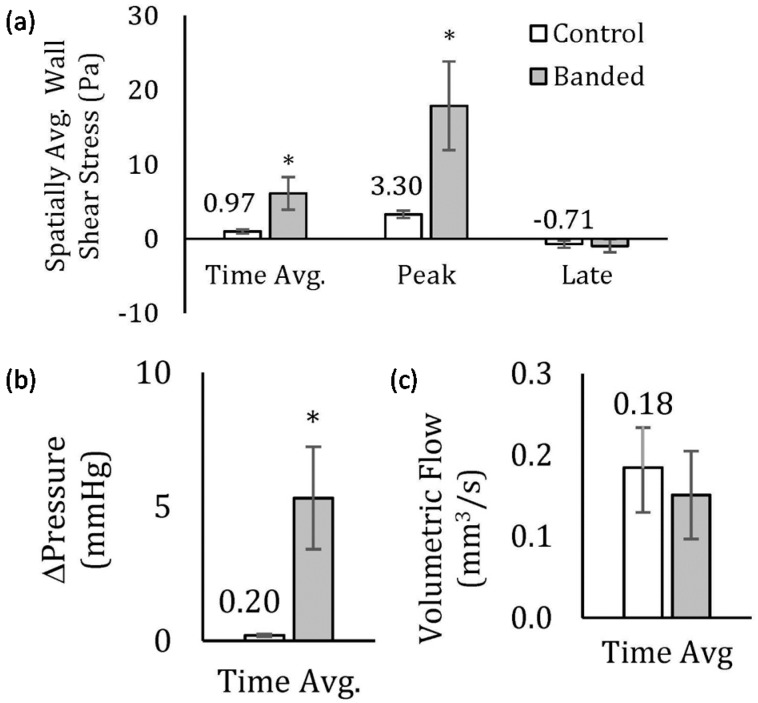
Hemodynamic variables, including (**a**) the spatially-averaged wall shear stress at the time-averaged flow condition, during peak flow conditions and late in the cardiac cycle during retrograde flow, (**b**) time-averaged pressure gradient and (**c**) the volumetric flow rate. * *p* < 0.05.

When spatially averaged, significant differences were found between the time-averaged control (0.97 ± 0.26 Pa) and banded (6.1 ± 2.19 Pa) wall shear stresses (*p* = 0.002) (Figure 6a). Peak spatially-averaged wall shear stresses were also found to be significantly different between control (3.30 ± 0.50 Pa) and banded (17.8 ± 5.96 Pa) hearts (*p* = 0.002) (Figure 6a). No statistical differences in shear stresses were found at the late retrograde flow condition (control = −0.71 ± 0.49 Pa, banded = −0.95 ± 0.88 Pa; *p* = 0.32). Banding generated a 5.32 ± 1.19-mmHg pressure gradient across the banding site, whereas normal hearts experienced a pressure drop of 0.20 ± 0.05 mmHg through this section (Figure 6b) (*p* < 0.001). Overall, no statistical differences were found between the volumetric flow rates in the control (0.18 ± 0.05 mm^3^/s) and banded (0.15 ± 0.05 mm^3^/s) embryonic chick hearts (*p* = 0.32) (Figure 6c).

### 3.4. Effect of OFT Banding on Relative Expression of Genes Involved in Valve Development

Real-time PCR was carried out to investigate the effects of constricting the OFT on the expression of a selected panel of genes that are critical to valve development (Table 2). Transcript levels of the mechanotransducer *rhoA* were significantly lower in the OFT from banded hearts relative to OFT of control hearts (*p* = 0.02). No significant differences were observed in the mRNA expression of shear-responsive *klf2* between OFT of banded and control hearts (*p* = 0.12).

The OFT of banded hearts exhibited significantly decreased mRNA levels of *collagen1α1* (*p* = 0.01) and increased transcript levels of *periostin* (*p* = 0.01) compared to OFT tissue of the control hearts. Though there was a downregulated trend in the expression of *tenascin C* (*p* = 0.08) in OFT from banded hearts, there were no significant differences in the mRNA levels of *elastin* (*p* = 0.19) and *vinculin* (*p* = 0.12) in the OFT of banded *vs.* control hearts.

The expression of *TGFβRIII* mRNA and that of *TGFβ3* were significantly upregulated (*p* = 0.004 and *p* = 0.02, respectively) in OFT of banded *vs.* control hearts; however, there were no differences in the expression levels of *TGFβ2* (*p* = 0.25) in OFT tissue from hearts in these two groups. Moreover, no significant differences were observed in the transcript levels of *snai2* (*p* = 0.14) and *has2* (*p* = 0.1), while mRNA expression of *mmp2* was significantly upregulated (*p* = 0.03) in the OFT from banded hearts compared to OFT tissue from controls. Though the mRNA expression levels of *CDH11* were significantly downregulated (*p* = 0.02) in OFT of banded hearts, there were no significant changes in transcript levels of *filamin A* (*p* = 0.34) between OFT of banded *vs.* control hearts.

**Table 2:** Differential expression of genes critical to valve development, upon OFT banding, in OFT tissue of banded and control hearts. * *p* < 0.05.

Gene	OFT from Control Hearts	OFT from Banded Hearts	*p*-Value
**Shear-/Flow-Responsive**			
*rhoA*	1.00 ± 0.08	0.88 ± 0.04	0.02 *
*klf2*	1.00 ± 0.24	0.83 ± 0.07	0.12
**ECM**			
*collagen1a1*	1.00 ± 0.23	0.60 ± 0.06	0.01 *
*periostin*	1.00 ± 0.36	2.15 ± 0.38	0.01 *
*tenascinC*	1.00 ± 0.20	0.80 ± 0.12	0.08
*elastin*	1.00 ± 0.32	0.83 ± 0.12	0.19
*vinculin*	1.00 ± 0.07	1.07 ± 0.06	0.12
**EMT/Cell Migration**			
*TGFβRIII*	1.00 ± 0.12	1.28 ± 0.05	0.004 *
*TGFβ2*	1.00 ± 0.18	0.92 ± 0.10	0.25
*TGFβ3*	1.00 ± 0.20	1.34 ± 0.13	0.02 *
*snai2*	1.00 ± 0.47	0.72 ± 0.09	0.14
*has2*	1.00 ± 0.04	0.95 ± 0.05	0.10
*mmp2*	1.00 ± 0.05	1.54 ± 0.27	0.03 *
*CDH11*	1.00 ± 0.12	0.77 ± 0.09	0.02 *
*filamin A*	1.00 ± 0.23	0.97 ± 0.09	0.34

## 4. Discussion

Valvular disease is the most common type of CHD. Disease conditions due to abnormal cardiac valve structure and function, including stenosis and regurgitation, may occur congenitally, but may be symptomatic only in adulthood. Consequently, replacement of diseased heart valves is the second most common heart surgery in the U.S., with a majority of the replaced aortic valves exhibiting congenital abnormalities [18]. This underscores the importance for understanding the mechanisms underlining the development of cardiac valves.

The fact that hemodynamics strongly influences heart valve development has been established by several models of altered blood flow, resulting in abnormal valves and defective septa [19,20,21,22,23,24]. It is well established that EMT is vital in the development of heart valves; however, it is not clear how hemodynamics regulates the EMT process. The present study aims to understand the role of blood as a physical factor in the developmental process of OFT valves at the embryonic stage.

Hemodynamics in the chicken embryonic heart were altered by banding the OFT at the OVJ. This constriction led to a significant “jet effect” at the OVJ, as revealed by ultrasound-mediated flow velocity measurements and prolonged increased levels of wall shear stress. Despite the increased cardiac afterload, the volumetric flowrate and heart rate were similar between controls and banded hearts. This result is indicative of adequate distal tissue perfusion and little change to systemic cardiovascular physiology. A subsequent lumen narrowing was also experienced in the distal portions of the OFT and confirmed by AMIRA 3D reconstructions. This distal lumen narrowing is consistent with the findings of other researchers [25], who hypothesized that this effect may be due to paracrine factors released at the banding site. Regardless, at a consistent volumetric flow, the narrowed lumen led to an increase in spatially-averaged wall shear stresses and a concatenate increase in the differential pressure. Flow-induced wall shear stresses are a result of the viscous properties of flowing blood, thereby creating physical signals that are sensed directly by the epithelial cell-lined lumen. The present work focuses on the effects of alteration in shear stresses; however, pressure and the physical constraints imposed by the banding model could play a pivotal role in the EMT process and the differentiation, morphology and gene expression effects observed herein. The role of these loads and the physical constraints are reserved for future work.

The control peak flow velocities measured for the OFT in our study (8.57 ± 1.28 cm/s) compare well to those of prior researchers, including Bharadwaj *et al.* for HH16 (9.2 ± 0.09 cm/s) and Liu *et al.* for HH18 (6.2 ± 0.7 cm/s) [26,27]. Liu *et al.* also reported a retrograde flow velocity of −2.0 ± 2.0 cm/s, and we found this value to be very close at −0.72 ± 0.77 cm/s. An estimation of time-averaged velocities from the full velocity profile of both of the aforementioned studies is also in the range of our measured value of 2.54 ± 0.68 cm/s. Our banding model, however, has Doppler measured centerline velocities much greater than those presented by Midgett *et al.* [28]. Despite those differences, our results are reasonable when considering proportional changes in cross-sectional area caused by the banding process.

Spatially-averaged wall shear stresses were determined using computational fluid dynamics simulations of 3D reconstructed hearts. CFD, rather than analytical approaches, was employed to enable the implementation of realistic geometries that allow for out-of-plane rotations and changes in cross-section shape. Although analytical solutions provide a good estimate of developed blood flow behavior in a circular cross-section, we observed that actual geometries, especially around the OFT, were non-uniform. The reconstruction and smoothing process can lead to undesired errors in sensitive hemodynamic properties. Despite this, we found time-averaged (0.97 ± 0.26 Pa) and peak (3.30 ± 0.50 Pa) wall shear stress values to be near those found by Bharadwaj *et al.* for the HH16 chick (0.36 Pa and 0.97 Pa, respectively) [26]. Liu *et al.* also showed graphically the peak wall shear stress for the HH18 chick to be somewhere around 6 Pa [27]. Our values fall between these two estimates, and the differences could be attributed to the source tissue location, assumptions on the velocity profile, tissue fixation geometry or from spatially averaging across a larger tissue area. Without more advanced imaging modalities (e.g., micro-CT), we are unable to quantify cushion expansion in real time and require tissue fixed under physiological conditions for our geometric-based CFD analysis. These two factors contribute to error in our shear stress calculations and are acknowledged limitations to this work. Regardless, the results of our CFD study provide a reasonable comparison between control and banded tissues.

Cushion volume and number of cells undergoing EMT were decreased by 39% and 69%, respectively, in the OFT of the banded hearts relative to control hearts. These observations highlight the fact that hemodynamics plays a significant role in the formation of the valve primordia during the initial stages of valve development that occur in HH16–17 embryos, the stage chosen for the OFT banding surgery. These reductions in OFT cushion volume and cell number could potentially lead to the formation of abnormal, hypocellular OFT valves.

As evidenced in the CFD model, there is a significant change in wall shear stress in, and distal to, the banding regions. However, for technical reasons, cushion tissues (including the myocardial sleeve) of the entire OFT were collected, which includes regions directly beneath/surrounding the band, as well as regions post- and pre-constriction. Thus, the gene expression results reported in this study represent changes that occur in the whole OFT tissue rather than at discrete locations. Since CFD simulations predicted spatially-averaged wall shear stresses to be significantly higher in the banded model, we compared the entire tissue gene expression to the entire tissue shear stresses. Our future work will focus on the local effects of shear stresses on markers of gene expression.

Endocardial cells, which line the lumen of the embryonic heart and the adult cardiovascular system, are the first to experience and respond to shear stress by mechanotransduction signaling pathways. When comparing flow (therefore, shear stress) to no flow (no shear), we have previously shown that *rhoA*, a small GTPase involved in regulating the actin cytoskeleton, is upregulated in the presence of flow in *in vitro* cultures of HH25 AV [13] and OFT [14] cushions and is also an important mechanotransducer, in 3D-cultured AV cushion explants [13]. At high levels of shear stress, however, *rhoA* was shown to be downregulated compared to physiological levels [14]. In the present study, we also observed a significant decrease in *rhoA* transcript levels in OFT of banded hearts. This decrease in *rhoA* transcription could potentially lead to inappropriate mechanotransduction and, thus, altered ECM production, leading to abnormally-formed OFT valves. Krüppel-like factor 2 (klf2) is an important shear-responsive transcription factor [29,30], appears to have a role in normal valve development and regulates the EMT process [31]. We did not observe any significant change in the mRNA expression of this gene upon constricting the OFT. This may be a consequence of analyzing the expression of this gene in whole OFT and not just the region where shear stress is altered.

The production/deposition of fibrous ECM proteins is important to maintain valvular integrity in order to allow valves to function efficiently and to prevent the backflow of blood during the cardiac cycle; consequently, aberrant expression and deposition of valve ECM proteins is associated with abnormalities in valve development and pathological states [18]. Collagen is the most abundant fibrous ECM protein in the mature valve, with mutations in collagen1α1 leading to, among other conditions, aortic valve insufficiency that requires valve replacement [18]. Tenascin C is a matricellular protein that is highly expressed during development and is associated with endocardial cushion tissue EMT. Elastin is found in the ventricularis, the aspect of the OFT valves that faces blood flow. It has been shown, in humans, that elastin content increases in the outflow valves from the fetal stage to adult [32]. In our study, we found a decreased expression of *collagen1α1* at the mRNA level and a downregulated trend in mRNA expression of *tenascinC* in OFT from banded hearts relative to that from controls. We have previously shown that in the presence of flow and, therefore, shear stress, transcript levels of *collagen1α1* and *tenascin C* are decreased in *in vitro* 3D-cultured HH25 OFT cushions compared to no flow controls [14]. Yet, when compared to physiological levels of shear stress, there were no observable differences when shear stresses were elevated to pathologically high levels (e.g., those generated from OVJ banding) [14]. The reduction in *collagen1α1* may be attributed to the change in shear stress through the banded OFT. However, it appears that spatially-averaged shear stress does not significantly influence expression of *elastin* and *vinculin*, at least at the mRNA level, at this embryonic stage. Importantly, it should be pointed out that the presence of flow did decrease mRNA expression of *elastin* in 3D-cultured OFT cushions from HH25 hearts and that pathologically high levels of shear stress also increased mRNA expression [14]. These differences are also likely attributable to the stage-dependent influence of hemodynamics on the transcription of this gene. In the present study, we observed an increase in transcripts of *periostin* in the high shear stress model, which are in agreement with our *in vitro* 3D OFT explant experiments [14], where transcript levels of *periostin* were upregulated in OFT cushion tissue. Periostin is a product of TGFβ3 signaling and has been shown to regulate chick AV valve maturation [33].

In the current study, we investigated the expression of a representative panel of genes important for EMT. The type III TGFβ receptor (TGFβRIII) is the ligand-presenting receptor and plays an important role in cushion EMT [34,35]. We observed a significant increase in gene expression of *TGFβRIII* and *TGFβ3* in the OFT tissue of banded hearts. However, there was no significant difference in expression levels of *TGFβ2* between OFT of the banded and control hearts. Snai2 is an important transcription factor of TGFβ signaling that induces EMT by decreasing the expression of adhesion molecules, including E-cadherin [3]. Even though we did not observe any alteration in gene expression of *Snai2* in OFT tissue from the banded hearts, there was a significant decrease in the number of cells undergoing EMT and, thus, invading the cushion tissue. This may be due to the effect of altered hemodynamics on a Snai2-independent EMT process. Thus, there seems to be activation of only the upstream events of TGFβ signaling in OFT tissue by the change in hemodynamics in the heart. The increase in *TGFβ3* levels could partially contribute to the enhanced expression of *periostin* in OFT tissue of the banded heart. Cadherin-11 (CDH11) is a type II classical cadherin that is found in the endothelium and mesenchyme of embryonic cushion tissue and may play a migratory role in populating the cardiac jelly [36]. OFT banding led to a significant downregulation in the expression of *CDH11* in OFT tissue. This indicates that changing cardiac hemodynamics in the embryonic heart could potentially affect the migration of endothelial cells, an important process of valve formation. Lastly, we found a significant increase in the expression of matrix metalloproteinase 2 (*mmp2*) in the OFT of banded hearts. MMP2 is required for degradation of the ECM and cell migration. However, it should be noted that mmp2 has to be proteolytically activated [37], which was not investigated in our study. Thus, we can only conclude that altered intracardiac hemodynamics seems to influence the expression of this gene at the mRNA level. These gene expression studies support the central theme that altered hemodynamics regulate critical valve gene expression. Moreover, using this new *ex ovo* model, we can continue to examine the role of hemodynamics and begin to correlate changes with corresponding cellular responses *in vivo*. Something to this point could not previously be achieved.

## 5. Conclusion

In summary, altered hemodynamics led to changes in OFT cushion volume, the number of invading mesenchymal cells in the OFT cushions and the expression of genes involved in ECM development, EMT signaling and cell migration. The present study, thus, contributes to the field of valvulogenesis and highlights the importance of normal hemodynamics in proper valve development.
